# Sample preparation and processing

**DOI:** 10.1155/S1463924691000135

**Published:** 1991

**Authors:** T. D. Geary

**Affiliations:** Division of Clinical Chemistry Institute of Medical and Veterinary Science Box 14 Rundle Mall Post Office Adelaide South Australia 5000 Australia

## Abstract

Errors made during the sample preparative process contribute significantly to the total analytical error of immunoassays. Reduction in the magnitude of these errors have been made by improvements in the manualfluid handling systems and mechanization of the individual steps in the process. The mechanization has been stimulated by developments in both computing hardware and software. A laboratory's workflow and workload will indicate the degree of mechanization required.


Journal of Automatic Chemistry, Vol. 13, No. 2 (March–April 1991), pp. 57–58

### Sample preparation and processing

**T. D. Geary**
*Division of Clinical Chemistry, Institute of Medical and Veterinary Science, Box 14, Rundle Mall Post Office, Adelaide 5000, South Australia, Australia*

*Errors made during the sample preparative process contribute significantly to the total analytical error of immunoassays. Reduction in the magnitude of these errors have been made by improvements in the manual fluid handling systems and mechanization of the individual steps in the process. The mechanization has been stimulated by developments in both computing hardware and software. A laboratory's workflow and workload will indicate the degree of mechanization required.*

This paper concentrates upon sample preparation and processing. The important linking role played is illustrated in figure 1.

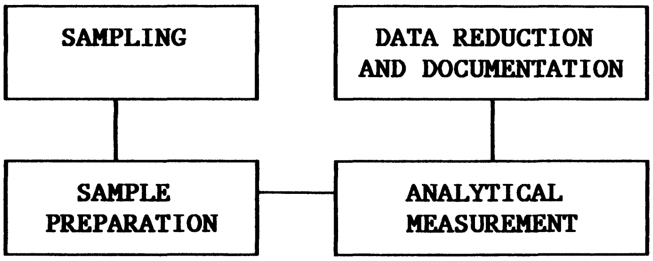

*Figure 1.*

'Sample preparation' includes those necessary steps prior to the measurement process and includes:

(1) Extracting, dissolving, suspending or modifying an analyte so it is suitable for the measurement process.
(2) Removing interferents.
(3) Adding calibration and other materials used for monitoring the system.

There have been four basic approaches taken. Firstly, there are the manual fluid handling devices. Secondly, a mechanized unit can be used with manual transfer to the reading unit. The process can be further simplified by mechanization of the transfer step, and, finally, a discrete robot can be used to perform each step in the immunoassay process.

Sample preparation has been seen as the weak link in the mechanization of immunoassay. It represented a major source of errors due to the manual steps involved, it was labour intensive, expensive, time-consuming, slow and disconnected from the automated flow of information. In some situations, it exposed people to hazardous environments, sensitive equipment to manual interference, and caused problems with training and retraining. This has now changed with a large number of manufacturers offering immunoassay systems, each with varying degrees of mechanization.

The method used for the mechanization of sample processing is dictated to a large degree by two factors: the complexity of the task and the volume of work to be performed. Manual techniques are suitable for low volume, and, generally, to low complexity of the task at hand. Variable mechanization, where the procedure steps are programmed at the beginning of each batch, is cost effective for medium workload with low to high complexity of sample preparation. Fixed program analysers and dedicated robots are effective when handling high sample volumes with a range of processes with varying complexities.

The manual techniques have been assisted greatly by the use of pipettes, which can be air, liquid or positive displacement. Such liquid handling systems are diverse in design to assist with their adaptation to meet the requirements of various analytical procedures. The pipettors may be fixed or variable with respect to volume of dispensing; they may have single or multiple tips. All other sample preparative procedures rely upon the mechanization of the steps carried out in the manual procedure.

A mechanized sample processor could include the following:

(1) *Probe system*: This may be single or dual.
(2) *Pump*: The pump may be peristaltic or controlled by an electronically controlled stepper motor.
(3) *Pipette-washing procedure*: The carry-over from sample to sample, or reagent to reagent, has been improved by the flexibility of the process.
(4) *Sample carousel*: The sample can be identified by using bar codes and either a light pen or a bar-code reader on the analyser.
(5) *Washing system*: A washing procedure is available for the reaction vessel whether this is a microtitre plate or test-tube.
(6) *Reagent dispenser*: There are a variety of nozzle heads, pump and syringe arrangements used to dispense the reagent. The pipetting system uses a liquid-level detector.
(7) *Magnetic stirrer*: The stirrer/mixer when used for reagents stops before the reagent probe enters the container.
(8) *Computing capacity*: The system has considerable computer storage capacity, special software functions and step by step programming.

0142–0453/91 $3.00 © 1991 Taylor & Francis Ltd.

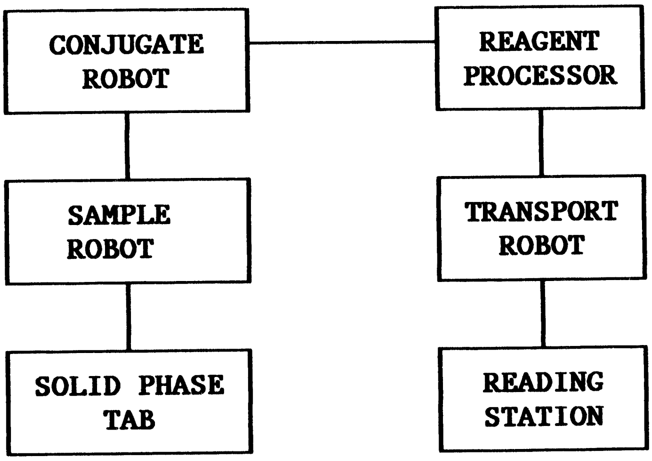

*Figure 2.*

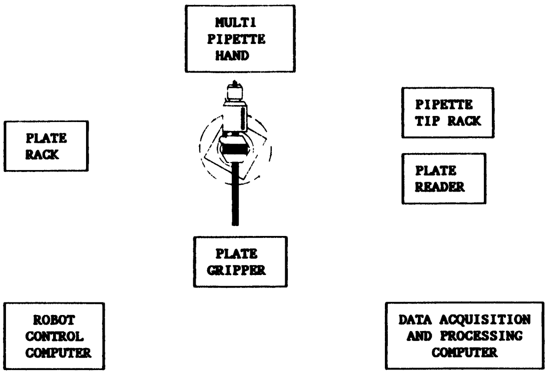

*Figure 3.*

The development and subsequent widespread use of the microchip has been the major impetus for the evolution of mechanized systems. This development, in association with refinements in programming, have led to the use of robots as part of mechanized systems. This is very evident in the general routine laboratory where the impact of the microchip and various programming languages has led to the demise of most manual techniques.

The development of robotics as a science has depended upon the use of microchip technology, advances in programming and the use of specialized commercial software. The development has led to the use of robots in mechanized and automated non-isotopic immunoassay systems, with the closing of the segments between sample processing through transfer of product to reader with data reduction and display. There are a number of such mechanized processors on the market, and the mechanized operation of one system is used as an example in figure 2.

In addition to the foregoing mechanized approach to immunoassay, a cylindrical robot can be used to give greater flexibility and to meet varying programming needs (figure 3).

A robot can be defined as follows:

> An automatically controlled, reprogrammable, multipurpose, manipulative instrument with several degrees of freedom, which may be either fixed on place or mobile for use in an automated application.

Robotic systems can prepare samples, control reactions, calculate results, order repeats, perform repeats and produce a hard or soft display.

There have been significant advances made in the area of sample processing so that each of the four approaches: manual, independent processor, integrated total system and dedicated robots, have seen progress. The most appropriate approach is decided in the selection process as part of the preliminary definition of a laboratory's requirements. As an example, the author's laboratory recently undertook an assessment of a number of the commercial systems available and the conclusion was that with our current workflow patterns a manual system continues to be the most cost effective. So the clear definition of requirements remains one of the most important determinants when considering the introduction of mechanization of immunoassay.

